# Advances of Recombinant Adenoviral Vectors in Preclinical and Clinical Applications

**DOI:** 10.3390/v16030377

**Published:** 2024-02-28

**Authors:** Luca Scarsella, Eric Ehrke-Schulz, Michael Paulussen, Serge C. Thal, Anja Ehrhardt, Malik Aydin

**Affiliations:** 1 Department of Anesthesiology, Center for Clinical and Translational Research, Helios University Hospital Wuppertal, Witten/Herdecke University, 42283 Wuppertal, Germany; serge.thal@helios-gesundheit.de; 2 Virology and Microbiology, Center for Biomedical Education and Research (ZBAF), Department Human Medicine, Faculty of Health, Witten/Herdecke University, 58453 Witten, Germany; eric.ehrke-schulz@uni-wh.de (E.E.-S.); anja.ehrhardt@uni-wh.de (A.E.); 3 Laboratory of Experimental Pediatric Pneumology and Allergology, Center for Biomedical Education and Science (ZBAF), Department of Human Medicine, Faculty of Medicine, Witten/Herdecke University, 58453 Witten, Germany; 4 Chair of Pediatrics, University Children’s Hospital, Vestische Kinder- und Jugendklinik Datteln, Witten/Herdecke University, 45711 Datteln, Germany; michael.paulussen@uni-wh.de; 5 Institute for Medical Laboratory Diagnostics, Center for Clinical and Translational Research, Helios University Hospital Wuppertal, Witten/Herdecke University, 42283 Wuppertal, Germany

**Keywords:** adenovirus, adenovirus biology, viral vector, vaccine, oncolytic, gene therapy, cancer therapy

## Abstract

Adenoviruses (Ad) have the potential to induce severe infections in vulnerable patient groups. Therefore, understanding Ad biology and antiviral processes is important to comprehend the signaling cascades during an infection and to initiate appropriate diagnostic and therapeutic interventions. In addition, Ad vector-based vaccines have revealed significant potential in generating robust immune protection and recombinant Ad vectors facilitate efficient gene transfer to treat genetic diseases and are used as oncolytic viruses to treat cancer. Continuous improvements in gene delivery capacity, coupled with advancements in production methods, have enabled widespread application in cancer therapy, vaccine development, and gene therapy on a large scale. This review provides a comprehensive overview of the virus biology, and several aspects of recombinant Ad vectors, as well as the development of Ad vector, are discussed. Moreover, we focus on those Ads that were used in preclinical and clinical applications including regenerative medicine, vaccine development, genome engineering, treatment of genetic diseases, and virotherapy in tumor treatment.

## 1. A Historical Introduction into the Discovery of the Adenovirus

Following the first isolation of adenovirus (Ad) from human adenoid tissues in 1953 [1], it has been established that Ad can infect a wide range of vertebrates [2,3]. Due to the observation of its cytopathogenic effects in adenoid and tonsil tissues during prolonged cultivation, Ad was initially termed as an ‘adenoid degeneration agent’ abbreviated as an A.D. agent [1]. A year later, during an epidemic at Fort Leonard Wood, a microbial agent was identified in throat washings from patients with primary atypic pneumonia. Similar to the earlier cases, it was observed that this unidentified agent caused cytopathogenic changes in human cell cultures and was designated as the ‘respiratory illness (RI) agent’ [4,5].

In 1954, a small infectious outbreak of febrile pharyngitis with conjunctivitis was reported from a hospital, leading to the proposal of the term ‘adenoidal-pharyngeal-conjunctival agent’ [6]. Furthermore, in July 1956, an article published in the journal Science, highlighted the necessity of assigning a common name to this group of predominantly respiratory viruses. However, institutions worldwide participated in this discussion and, during a meeting on 25 May 1956 in New York, USA, it was decided to adopt the term ‘Adenovirus group’ [7].

## 2. Adenovirus Biology and Clinical Presentation

Adenovirus belongs to the family of Adenoviridae [8,9,10], specifically within the genus Mastadenovirus [11,12,13]. Human Ads are categorized into seven species (A to G), comprising 114 identified types (http://hadvwg.gmu.edu/ (accessed on 22 January 2024, [14,15,16]). These non-enveloped double-stranded DNA viruses present a pseudo T = 25 icosahedral capsid with a diameter of 95 nm (vertex to vertex) [17]. The virion consists of three major proteins, i.e., hexon, penton base, and fiber [18,19].

The hexon, which represents approximately 60% of the virion mass, is a highly conserved structure throughout the Adenoviridae family, composed of more than 900 amino acids [20,21,22]. The icosahedral capsid features a penton complex at its 12 vertices, comprised the penton base and fiber [18,23,24,25,26]. The penton base is a pentameric structure that fills the space at each vertex left by the five peri-pentonal hexons [22,27,28], while the fiber is an elongated protein divided into an N-terminal ‘shaft’ anchored on the capsid and a C-terminal knob that interacts with receptor structures [21,29,30]. Minor capsid components such as proteins IIIa, VI, VIII, and IX contribute to the capsid structure and stability [31,32]. Within the capsid, there are basic proteins (protein V, VII, and µ) that are packaged within the virion together with the genome [33,34,35,36].

Adenovirus utilizes several receptors for cell entry, including Coxsackie and adenovirus receptor (CAR) [37,38,39,40], CD46 [11,41], desmoglein-2 (Dsg-2) [13] Glycans GD1a [42] and polysialic acid [43], etc. Notably, Ad binding to soluble factor IX [44] and X [45,46,47] may facilitate cell entry.

The linear double-stranded DNA genome of Ads has a length of ~34 to 36 kilo-base-pairs (kb). At the ends, the Ad genome is flanked by highly conserved sequences defined as ‘inverted terminal repeats’ (ITRs). Downstream of the left ITR, a DNA sequence serving as a packaging signal (Ψ) is located, interacting with other viral proteins to encapsidate the Ad genome [48,49,50]. The coding region of the Ad genome contains four transcription units (E1, E2, E3, and E4) that encode early genes, as well as late transcription units (L1, L2, L3, L4, and L5) that are responsible for encoding late proteins [33,51,52,53,54,55,56,57,58,59]. The E1, E2, and E4 transcription units regulate the transcription and translation of late genes, which are crucial for the adenoviral replication [60]. In the region E1, two transcription units can be identified [61]. E1A proteins play an important role during initial steps of viral infection as they stimulate the transcription of viral genes [62,63]. Transcription unit E1A inhibits the transcription of certain cellular genes by inhibiting the homo- or hetero-oligomerization of p53 [64]. E1B assists with E1A to redirect the function of the host cell and suppresses apoptosis [65,66], thereby facilitating viral replication, and also contributes to the transformation of the host cell [67]. The E2 region can be divided into two segments: the proximal segment E2A and the distal segment E2B. Both segments encode proteins which are relevant for the replication of the viral genome [68]. The E3 region plays a critical role in the pathogenesis of the disease process by inhibiting both the specific immunity (i.e., cytotoxic T lymphocytes and CTL) and the innate immune response (i.e., tumor necrosis factor (TNF)-α) [69,70]. The products of the E3 region are not relevant for virus replication but can mediate the escape from the immune system of the host through inhibition of cytokine production, alteration of antigen presentation, and cellular apoptosis [71,72]. In addition, the E4 region encodes multiple proteins, that play an important role in maintaining the stability of viral RNAs during the later stages of infection [73]. The L1 to L5 transcription units code for the major structural proteins of Ad, including the major capsid proteins hexon, fiber, and penton [74,75,76].

**Figure 1:**
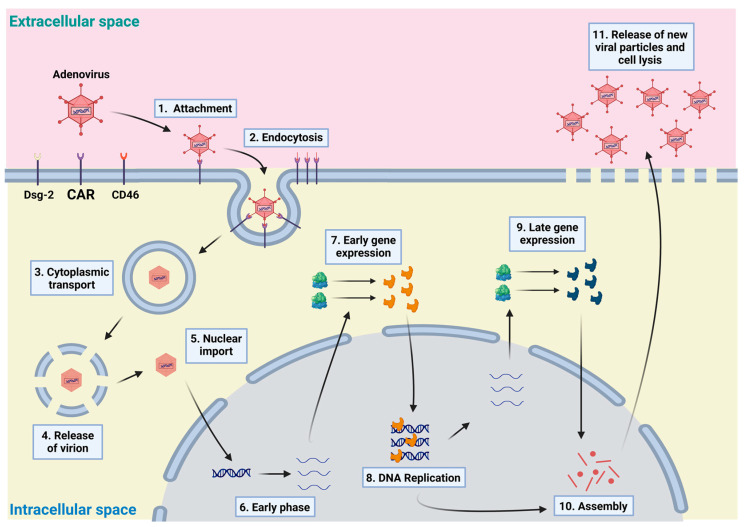
The life cycle of adenovirus. (1) Adenovirus (Ad) infection begins with the binding of Ad fiber to cellular receptors (e.g., CAR and CD46 or Desmoglein-2). (2) This binding activates the internalization of Ad through endocytosis. (3) During cytoplasmic transport, Ad presents proteins, which, in association with vesicular acidification, allow the release of the virion (4) near the nucleus. (5) The interaction with the nuclear pore complex facilitates the transport of the viral genome into the nucleus. In addition, the replication of Ad is divided into two stages: the early phase (6), during which genes are expressed that code for transcription factors. These factors regulate the expression of other viral genes as well as host cell genes, enabling the completion of the Ad life cycle. Subsequently, viral DNA replication (8) starts along with the expression of proteins encoded by late genes (9). This coordinated activity allows the assembly of new viral particles (10) and new viral particles are released through cell lysis (11). A detailed signaling cascade through virus entry is not presented in detail to preserve the overview. This figure was adapted from [89].

Furthermore, Ad is an obligate intracellular pathogen and needs the replicative and transcriptional apparatus of the host cell to enable replication and to continue its life cycle to form new viral particles [77,78]. As shown in Figure 1, following the binding of the adenoviral knob with the host cell surface receptors, the interaction between a highly conserved RGD motif penton base protein and an activated status of cellular integrins, such as α_v_β3/α_v_β5, may promote the Ad capsid internalization through clathrin-mediated endocytosis [79,80], while the proteins ‘fiber’ remain on the surface of the host cell [19]. There are also other internalization pathways, including lipid rafts, caveolin-mediated endocytosis, and macro-pinocytosis, that play a secondary role and are dependent on the type of the host cell [81]. During internalization, the Ad capsid vertex region (penton base, fiber, and peri-pentonal exons) disassembles and releases various proteins including the lytic protein VI, which mediate the vesicular membrane disruption [82] and the subsequent access of the capsid to the cytosol. Once in the cytosol, the leaky Ad capsid interacts with the dynein of the microtubular system of the cell. Subsequently, this interaction facilitates the passive transport of the Ad to the nuclear pore complex (NPC) and the hexon protein of Ad binds to the nucleoprotein Nup214 of NPC. The temporal interval between the initial interaction of Ad with surface receptors and the binding with NPC proteins is brief, lasting less than an h [82,83,84,85,86].

The interaction of Ad with NPC initiates a series of events, including priming, unlocking, and disruption of the virion. Ubiquitination of the Ad protein V results in the detachment of the adenoviral DNA from the protein complex, allowing the viral DNA to be transported into the nucleus [87,88]. This interaction between viral DNA and cellular replication promoters leads to the formation of viral replication compartments (VRCs). Importantly, the life cycle of Ad occurs extrachromosomally [89].

The assembly of new viral particles takes place within the nucleus and is synchronized with viral DNA synthesis [90] to facilitate adenoviral packaging [91]. The last step in the Ad life cycle involves the lysis of the host cell, which leads to the release of new virus particles. The mechanisms underlying lysis can vary and depend on both the specific virus and the characteristics of the host cell. For instance, Ad5 induces lysis through the overexpression of the adenovirus death protein (ADP) [92,93].

Regarding clinical manifestations, Ad infection is highly prevalent and exhibits a global distribution. Specifically, Ad can cause acute infections in the host [94] and can also lead to viral persistence [95]. It can be endemic, particularly in the pediatric population, where many Ad infections are diagnosed in children [96,97]. Certain populations, such as military recruits [98,99], patients with hematopoietic stem cell transplantation (especially allogeneic transplantation) [100], patients undergoing solid organ transplantation [101], individuals with congenital immunodeficiency [102], and those with acquired immunodeficiency [103], are more susceptible to Ad infections [97], associated with increased morbidity and mortality rates [101].

Moreover, Ad species D and E are predominantly linked to the development of keratoconjunctivitis [97], while species A and B can lead to respiratory, gastrointestinal, and urinary diseases [104,105]. Species C Ads are particularly associated with respiratory symptoms and they are the serotype most frequently isolated in children with severe acute respiratory infections [106]. Ad infections elicit both innate and adaptive immune responses. The initial response against Ad infection involves the release of different chemokines and cytokines, including interferon gamma (IFN-γ), TNF, interleukin (IL)-1, IL-2, and macrophage inflammatory proteins from non-antigen-specific cells, including dendritic cells and macrophages. In addition, natural killer (NK) cells are recruited and activated to restrict viral amplification [107,108]. Simultaneously, an antigen-specific response occurs, involving antigen-presenting cells (APCs) that present adenoviral antigens through major histocompatibility, which results in the release of cytokines by APCs, inducing T helper (TH1) (cellular) responses that activate cytotoxic processes of CD8^+^ T cells (through IL-2 and IFN-γ secretion), or TH2 (humoral) responses that stimulate the production of antigen-specific antibodies against adenoviral antigens (through IL-4 release) [109]. The cellular and humoral response of the host act to constrain virus infection and replication.

Moreover, Ad can also lead to latent infection and persist in various tissues, such as myocardiocytes [110], or in lymphatic niches (e.g., tonsils, adenoids, and intestinal lymphatic tissue) [111,112]. This persistence of Ad leads to the question of whether latent Ad infection may contribute to cancer development. Although Ad genome sequences are detected in some malignant tumors (e.g., cell lymphoma and glioblastoma [113,114]), studies have not yet demonstrated a direct relationship between Ad infection and tumorigenesis (summarized in [115]).

## 3. Adenovirus as a Vector and Its Production on a Large Scale

The intrinsic properties of Ads, including their episomal nature, minimize the risk for undesired insertional mutagenesis, eliminating the potential of germ-line transmission [116]. The Ad genome is well known and easily modifiable [117], allowing the production of recombinant replication-competent [118] and replication-defective vectors [119]. With a wide tropism and high transduction capacity for both replicating and non-replicating cell types [120], Ads are excellent gene delivery tools due to their simple manipulation of their genome [121,122].

**Figure 2:**
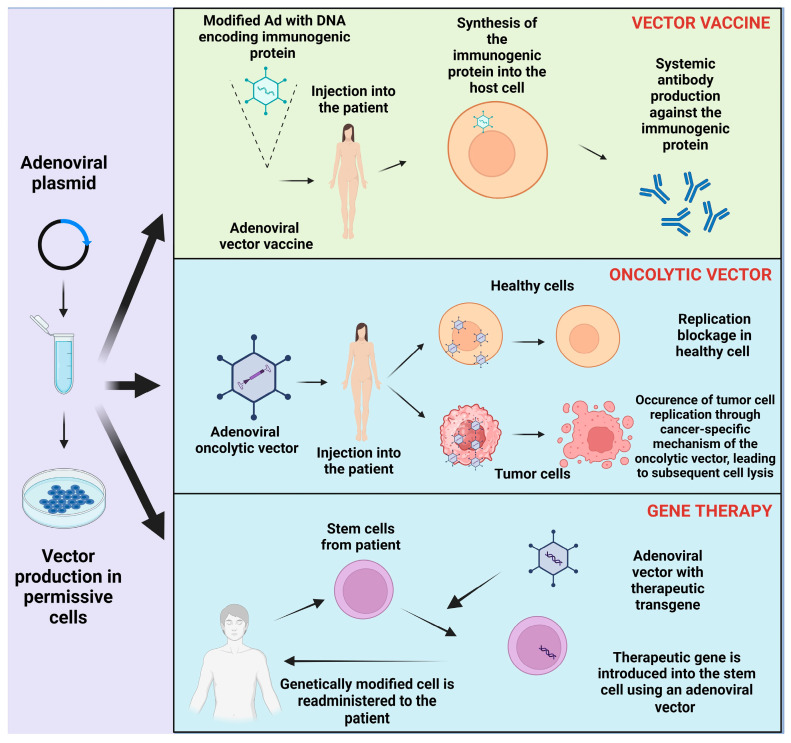
Versatile clinical applications of adenoviral vectors in vaccine design, oncolytic and gene therapy. Adenovirus-mediated gene delivery can be used for vaccine development (as Ad can deliver genes coding for immunogenic proteins into the host, which trigger the development of immunity in the host) and for oncolytic therapy (targeted therapy, which consists of administering recombinant Ads, leading to tumor cell-specific replication of recombinant Ad only in tumor cells and their consequent lysis) [131,132,133].

Another advantage of Ads is their strong immunogenicity [123], making them suitable for vaccine development. Figure 2 illustrates the clinical application of adenoviral vectors. So far, there are three generations of adenoviral vectors. The first-generation vector type presents the replication-defective (or incompetent) Ads, which serve as vectors in vaccine production or gene therapy. Replication-defective Ads imply that the virus cannot proliferate uncontrollably or assemble into infectious particles [119]. In these first-generation Ad vectors, the E1 region typically contains a partial or complete deletion, resulting in the loss of expression of the essential replication proteins E1A and E1B. The deletion of E1 sequences in first-generation Ad vector transgenes allows the insertion of transgenes into the E1 region. To further increase the transgene insertion capacity, the E3 region can also be deleted, allowing the insertion of transgenes of up to 8.2 kb. However, the limited duration of cargo and leaky expression of viral genes leading to immune responses are the challenges associated with first-generation Ad vectors [124].

To enhance the capacity of the vector for transporting foreign DNA (up to 14 kb), additional deletions in the E2 and E4 regions, beyond those in the E1 and E3 deletions, were introduced, characterizing the second-generation of vectors [125,126]. These vectors exhibit reduced immunogenicity compared to first-generation vectors and a lower likelihood of generating replication-competent Ads (RCA). However, the production of Ad vectors can also be associated with issues, such as the difficulty in establishing stable cell lines for production. This complexity is inherent to the second-generation vector and resulted in low viral titers and reduced gene expression [127].

To address these challenges associated with first- and second-generation vectors, the majority of adenoviral coding regions for viral proteins, excluding cis-acting elements required for vector genome replication, such as ITR sequences and encapsidation, were deleted and replaced by foreign DNA and transgenes of interest [127,128,129].

These modifications have significantly enhanced the transgene carrying capacity to up to 36 kb and have reduced the immunogenicity of these vectors. Consequently, they are safer for patient applications, and the duration of transgene expression can be extended. This development represents the third generation of Ad vectors, the so-called gutless, gene-deleted, or high-capacity adenoviral vectors [122]. The deletion of viral protein-coding sequences in these viruses makes them dependent on a helper virus (HV). Therefore, they are also characterized as helper-dependent Ads (HD-Ad). The HV must be present within the vector-producing cell, providing the necessary proteins for replication and packaging of the HD-Ad genome to the HD-Ad [128,129,130].

For the construction of Ad vectors, there are currently two different methods in use. The direct insertion of the foreign gene into the viral genome entails using a restriction enzyme [123] and homologous recombination between two plasmids (genomic and shuttle plasmids) either in a permissive cell line or in bacteria [134,135]. The first method, also defined as the in vitro ligation method, involves the direct insertion of the gene of interest into the Ad genome and is commonly used when inserting large DNA fragments [136,137]. On the other hand, homologous recombination is the most widely used and efficient method for the production of viral vectors. It involves the co-transformation of *Escherichia coli* such as BJ5183 that expresses nucleases [138] and thus is capable of homologous recombination. The thus obtained recombinant Ad (with the gene of interest in its genome) can be used for the transfection of producer cells [139]. As described, adenoviral vectors feature deletions in the E1 regions (first-generation), E2 and/or E4 (second-generation), or all coding regions (third-generation) to allow viral replication and production. To compensate for these deletions, modified cell lines are engineered to express the products of the missing regions of the adenoviral genome. For the generation of first-generation Ad vectors, human embryonic kidney (HEK) 293 cells were created by inserting the E1a and E1b sequences into their chromosome 19 [140,141]. Furthermore, the 911 cell line was generated based on the insertion of an E1 sequence of Ad5 into human embryonic retinoblasts (HER) cells [142] and lung adenocarcinoma-derived A549 cells were also used as producer cells after the insertion of E1 sequences [143]. The potential generation of RCA and a poor production of viruses are the common problems with the use of these cell lines [144]. For the second-generation vectors, cell lines deriving from HEK293, 911, and A549 cells with inserted E2 and/or E4 are used [145,146]. However, a problem arises due to the toxicity of the E2a and E4 proteins to the host cell [147]. The 293-C2 cells, which express E2a, have a viral production rate 30 times lower than the wild-type E2 vector [148]. Consequently, an E2T cell line was generated with intracellular expression levels of E2A regulated by tetracycline-controlling elements, reducing toxicity and enabling a yield similar to the wild-type E2 vector [146]. The VL2-20 and VK10-9 cells, containing the entire E4 sequence regulated by the dexamethasone-inducible MMTV promoter, are used for the production of E4-deleted adenoviral vectors [145]. For the production of third-generation vectors, not only is a suitable producer cell line required but a complementing Ad vector (helper vector) also provides packaging proteins to the viral particles being produced [149]. Through the expression of a Cre recombinase in the producer cells, encapsidation of the helper virus DNA can be prevented, ensuring that only the viral vectors can be produced [150,151].

Through the use of producer cells, the primary stock of the viral vector is created and subsequently amplified to obtain an adequate quantity of Ad vector in clinical and preclinical applications [152]. To be used in clinical settings, the Ad vector must be purified using methods such as cesium chloride density gradients [153], combined with ultracentrifugation [154], which is the most commonly used method to separate Ad vectors from other cellular debris. Using bioreactors containing continuous cell lines such as PER.C6 [151,155], a large scale production of Ad vector can be carried out. The subsequent purification involves chromatography-tandem ultracentrifugation or sequential chromatography [156].

## 4. Clinical Application

One of the first clinical applications of the Ad dates back to the 1950s, when live Ad4 and Ad7 were used as oral vaccines in North American military recruits to prevent respiratory disease (summarized in [157]). In 1989, the first in vivo gene transfer was demonstrated by a recombinant replication-deficient Ad with deleted E1 and E3 regions coding for β-galactosidase [158]. It was shown that Ad was effective in transferring genes in patients with hereditary α1-antitrypsin deficiency and cystic fibrosis [159]. Today, the potential clinical applications of Ad-mediated gene delivery range from regenerative medicine, vaccine development, and anticancer therapy up to gene therapy for monogenic diseases [124]. The following chapters provide an overview of these applications.

### 4.1. Regenerative Medicine

In 2006, Dr. Heather Greenwood defined regenerative medicine as an emerging interdisciplinary field of research and clinical applications. This field concentrates on the repair, replacement, or regeneration of cells, tissues, or organs, aiming to restore impaired function due to several causes, including congenital defects, diseases, trauma, and aging [160,161].

Within regenerative medicine, viral vectors find applications through two primary delivery approaches: in vivo [158] or ex vivo delivery [162]. In the first case, the viral vector is directly introduced into the patient to reach the targeted site of action. In the latter, the viral vector is inserted into target cells in a laboratory setting and treated cells are subsequently inserted to the site of damage [163].

In the context of Ad-based gene therapy for bone regeneration, the gene encoding the osteoinductive bone morphogenetic protein 2 (BMP2) plays a role in osteogenesis. Ex vivo approaches use the recombinant Ad-BMP2 to transduce mesenchymal stem cells, which are then implanted into bone defects within a matrix consisting of polylactide granules (which serve as a depot for genetic constructs or matrices for cell attachment) and with platelet-rich plasma as a source of growth factors and a binding gel. This ex vivo approach demonstrated a higher osteo-inductive effect compared to in vivo approaches [163].

In a pig infarction model, myocardial injection of recombinant Ad-VEGF121 improved myocardial perfusion and wall thickening after four weeks [164]. Similarly, intra-myocardial administration in the peri-infarct area of recombinant Ad-VEGF165 induced angiogenesis in the border zone of myocardial infarction [165]. In addition, endovascular intra-myocardial administration of Ad-VEGF-D^ΔNΔC^ increased perfusion and the ejection fraction in the infarct border zone in a porcine myocardial infarction model [166].

### 4.2. Adenoviral Vector Vaccine

The use of an adenoviral vector in vaccine design offers several advantages. Adenovirus can activate the innate immune system without the need for adjuvant substances [167]. The interaction of Ad with both surface and intracellular host proteins, such as integrins, pattern-recognition receptors, toll-like receptors (TLR), such as TLR-2, TLR-4, and TLR-9 [168], lactoferrin [169], and MyD88 [170], moderately stimulates an innate immune reaction [167]. This moderate activation of the innate immune response is appropriate to activate adaptive immune responses to transgene Ad products without causing severe side effects due to the excessive release of pro-inflammatory cytokines [171,172].

The adaptive immune response induced by Ad includes not only the production of antibodies but also a robust activation of CTL. Ad vector can transduce both immune and non-immune cells [173]. When transgene expression occurs in non-immune cells, immunogenic products are released from the cells [172]. The uptake of these products by APCs leads to the production of various isotypes and subclasses of specific antibodies [174,175]. Moreover, intramuscular administration can also stimulate moderate levels of mucosal immunity activity [172,176] through the induction of T helper 17 cells, which migrate to the gut mucosa and stimulate the proliferation of mucosal antigen-specific CTL [177].

High seroprevalence to Ad5 in the general population represents the strongest obstacle for conventional Ad-based vector vaccines [178,179]. Neutralizing antibodies against Ad5 are specific for hexon, fiber, and rarely for penton base proteins. Strategies to overcome these challenges include the use of serotypes with low seroprevalence, such as Ad11, 26, 35, 48; the application of recombinant Ad with modified hexon proteins [180,181]; and the utilization of non-human Ad vectors [182]. Moreover, strategies to hide the surface antigens of Ad have been developed, including the use of polyethylene glycol [183] coats, liposome-encapsulated vectors [184], and vesicle encapsulation techniques [185].

One of the most notable Ads in vaccine development is during the previous COVID-19 pandemic [186]. Vaccines such as ChAdOX1 nCoV-19 [187,188,189], Sputnik V [190], and Ad5-nCOV [191] are Ad-based vector vaccines expressing the full-length spike protein of SARS-CoV-2, while Ad26.COV2-S [192] codes for a recombinant spike protein. Other Ad-based vaccines include Ad26.ZEBOV (encoding glycoproteins from Ebola, Sudan, Marburg, and Tai Forest viruses nucleoprotein) [193], Ad26.ZIKV.001 (inducing neutralizing antibodies against Zika virus) [194], Ad26.Mos.HIV (an Ad26-based vaccine) [195,196], and ChAdOx1 (a replication-incompetent Ad5-based vaccine expressing influenza virus antigens nucleoprotein (NP) and matrix protein-1 (M1) [172,197]. Recent clinical trials about the application of adenoviral vectors in vaccine production are listed in Table 1.

### 4.3. Adenoviral Vectors and Gene Editing

Adenovirus has also demonstrated a versatile role in the field of genome editing techniques. The clustered regularly interspaced short palindromic repeat (CRISPR) and its accompanying protein (Cas9) is a highly efficient and precise technique enabling scientists to modify the DNA of an organism [198]. This methodology includes two important components: a guide RNA to match the target gene and an endonuclease, which induces a double-stranded DNA break [199]. Although adeno-associated viral vectors (AAVs) play an important role [200], adenoviral vectors can also be used to transport all the components of the system CRISPR/Cas9 [201], facilitating an in vivo-knock-in approach in models [202].

Furthermore, efforts to perform gene editing in hepatocytes have been undertaken using the CRISPR/Cpf1 system [203]. However, the widespread seropositivity among the general population for Ad represents a significant limitation in the use of viral vectors in this field [124].

### 4.4. Oncolytic Adenovirus and Cancer Therapy

The use of recombinant Ad as an oncolytic vector represents a novel strategy in cancer therapy, increasingly recognized in clinical trials since 2001, when oncolytic Ad (OAd) Onyx-015 was employed as a therapeutic intervention for advanced pancreatic cancer [204,205]. A critical requirement for ensuring the safety and efficacy of this therapeutic approach is the replication of OAd exclusively in tumor cells while healthy tissue does not perfectly enable vector replication [206]. The prototype of Ad-mediated virotherapy was ONYX-015 [207], an OAd characterized by a deletion in the region coding for E1b protein. Typically, the E1b protein binds to and inactivates p53 to promote viral replication. However, OAd lacking the E1b gene can replicate in cancer cells that are deficient in p53 expression [207]. Another example is demonstrated by an OAd carrying inactivating deletions in the region coding for the E1a protein [208]. Normally, E1a interacts with the retinoblastoma (Rb) protein of the host cell to neutralize its inhibitory effect on E2F, a transcriptional activator that initiates the S-phase of the cell cycle [209]. Consequently, OAd vectors with deleted E1a can replicate in cancer cells, where the expression of the Rb protein is suppressed [210]. However, the absence of OAd receptors on the surface of certain tumor cells limits its applicability [204].

The use of oncolytic Ad5 from species C is limited to cancer cells with the CAR on their cell membrane. To address this limitation, utilizing group B Ad as oncolytic vectors, which present a higher affinity for the CD46 receptor, offers advantages. For instance, oncolytic Ad35 demonstrates a 100-fold higher activity than oncolytic Ad5 against breast cancer [211]. Similarly, Ad5 has a low efficacy in hematological malignancies, due to low expression of CAR on blood cells [212]. Despite the good affinity of oncolytic Ads of species D for CD46 and salicylic acid [213], their short fiber reduces the binding affinity to the Ad receptor, making them more suitable for vaccine development [214]. In addition, oncolytic Ads from groups E, F, and G, which primarily interact with CAR receptor, have not been successful in this therapeutic context [206].

The insertion of a transgene coding for adjuvant factors into the Ads genome can improve their therapeutic efficacy, acting synergistically with their oncolytic action [215]. Transgenes can be also inserted into the E3 region [216]. Either human cytomegalovirus promoters [217] or heat shock protein promoters [218] are designed to express the transgene. A further approach is to insert the transgene into a late gene, which has shown an advantage in terms of expression level and specificity [219]. Notably, armed Ad vectors carrying molecules such as GM-CSF [220], IL-12 [221], IL-15 [222], INF-γ [223], and TNF-α [224] have demonstrated significant success in clinical trials. A recent advancement involves the combination of armed OAd with chimeric antigen T-cell (CAR-T) therapy [225], as well as chimeric antigen receptor-NK cell therapy [206,226,227]. These applications show the potential for armed OAd to play a pivotal role in the future landscape of cancer treatment. Table 2 lists a few clinical trials of oncolytic adenoviral vectors.

## 5. Conclusions

Gene therapy using adenoviral vectors has been recognized as a promising therapeutic option for several clinical applications. The ongoing monitoring and analysis of outcomes among patients participating in clinical trials remain important for a thorough understanding of the long-term efficacy and safety of recombinant Ad vectors. The episomal nature of the adenoviral genome represents an important advantage over the commonly used recombinant lentiviruses in clinical settings due to the high transduction efficiency of Ads. However, despite these advantages, challenges remain. The widespread seroprevalence of Ads in the population presents issues that require careful consideration. In cancer therapy, where synergistic advantages have been observed, this includes the combination of armed oncolytic viruses and CAR-NK cell therapy [226]. Addressing these challenges and integrating therapeutic strategies at multiple levels is promising for advancing the field of gene therapy.

## Figures and Tables

**Table 1:** Application of an adenoviral vector in a vaccine clinical trial.

Disease	Phase	Clinical Trial Number
HIV	I	NCT00479999
Ebola	I	NCT02289027
HIV	I	NCT01989533
Malaria	I	NCT00371189
COVID-19 disease	II	NCT05027672
COVID-19 disease	I	NCT04568811
Prostata cancer	II	NCT00583752
Tuberculosis	I	NCT00800670
Hepatitis C	I	NCT01094873
Melanoma	II	NCT00010309

**Table 2:** Some selected clinical trials of oncolytic adenoviral vectors for anti-cancer therapy recruiting in the year 2023.

OAd	Transgene	Disease	Phase	Clinical Trial Number
Ad5-yCD/mut TKSR39rep- hIL-12	yCD, TK and hIL-12	Prostate adenocarcinoma	I	NCT02555397
Ad11p/Ad3	No	Solid epithelial tumors	I	NCT02636036
VCN-01/Ad5 based)	No	Solid tumors	I	NCT02045602
AdAPT-001	TGF-ß	Solid tumors	I	NCT04673942
Ad5/3-E2F-D24-hTNFa-IRES-hIL2 (TILT-123)	TNF-α, IL-2	Solid tumors	I	NCT04695327
OAdTILT-123	No	Melanoma	I	NCT04217473
Ad5-yCD/mut TKSR39rep-ADP		Astrocytoma	I	NCT05686798
VCN-01	No	Retinoblastoma	I	NCT03284268
Ad5-yCD/mutTKSR39rep-ADP	No	Pancreas Adenocarcinoma	I	NCT04739046
NG-641	FAP, CXCL9/ CXCL10/IFNa2	Solid epithelial tumors	I	NCT04053283

## Data Availability

Not applicable.
